# Delta-Like 1 Homolog (Dlk1): A Marker for Rhabdomyosarcomas Implicated in Skeletal Muscle Regeneration

**DOI:** 10.1371/journal.pone.0060692

**Published:** 2013-04-05

**Authors:** Louise H. Jørgensen, Jeeva Sellathurai, Erica E. Davis, Tania Thedchanamoorthy, Rua W. A. Al-Bader, Charlotte H. Jensen, Henrik D. Schrøder

**Affiliations:** 1 Department of Pathology, Institute of Clinical Research, SDU Muscle Research Cluster (SMRC), University of Southern Denmark and Odense University Hospital, Odense, Denmark; 2 Department of Clinical Biochemistry and Pharmacology, Laboratory of Molecular and Cellular Cardiology, Odense University Hospital and Department of Cardiovascular and Renal Research, Institute of Molecular Medicine, University of Southern Denmark, Odense, Denmark; 3 Department of Pediatrics, Center for Human Disease Modeling, Duke University Medical Center, Durham, North Carolina, United States of America; 4 Faculty of Veterinary Medicine, University of Liege, Liege, Belgium; University of Pecs Medical School, Hungary

## Abstract

Dlk1, a member of the Epidermal Growth Factor family, is expressed in multiple tissues during development, and has been detected in carcinomas and neuroendocrine tumors. Dlk1 is paternally expressed and belongs to a group of imprinted genes associated with rhabdomyosarcomas but not with other primitive childhood tumors to date. Here, we investigate the possible roles of Dlk1 in skeletal muscle tumor formation. We analyzed tumors of different mesenchymal origin for expression of Dlk1 and various myogenic markers and found that Dlk1 was present consistently in myogenic tumors. The coincident observation of Dlk1 with a highly proliferative state in myogenic tumors led us to subsequently investigate the involvement of Dlk1 in the control of the adult myogenic programme. We performed an injury study in Dlk1 transgenic mice, ectopically expressing ovine Dlk1 (membrane bound C2 variant) under control of the myosin light chain promotor, and detected an early, enhanced formation of myotubes in Dlk1 transgenic mice. We then stably transfected the mouse myoblast cell line, C2C12, with full-length Dlk1 (soluble A variant) and detected an inhibition of myotube formation, which could be reversed by adding Dlk1 antibody to the culture supernatant. These results suggest that Dlk1 is involved in controlling the myogenic programme and that the various splice forms may exert different effects. Interestingly, both in the Dlk1 transgenic mice and the DLK1-C2C12 cells, we detected reduced myostatin expression, suggesting that the effect of Dlk1 on the myogenic programme might involve the myostatin signaling pathway. In support of a relationship between Dlk1 and myostatin we detected reciprocal expression of these two transcripts during different cell cycle stages of human myoblasts. Together our results suggest that Dlk1 is a candidate marker for skeletal muscle tumors and might be involved directly in skeletal muscle tumor formation through a modulatory effect on the myogenic programme.

## Introduction

Dlk1 is a member of the Epidermal Growth Factor family [1]. It is expressed widely during development, but is detectable in only a few mature tissues including pituitary growth hormone cells, pancreatic beta cells and adrenal cortical cells [2]. Furthermore, Dlk1 has been observed in various carcinomas [3] including neuroendocrine tumors [4].

Dlk1 is transcribed from a single gene, which undergoes splicing to generate five alternative isoforms in addition to the full-length protein. Full-length isoform A and variant B both contain a protease-recognition site and can produce a soluble form of the protein whereas C, C2, D, and D2 all remain membrane-bound [1], [5]. It belongs to a group of imprinted genes, which have been associated recently with rhabdomyosarcomas but not other primitive childhood tumors [6], thus Dlk1 might be involved in skeletal muscle tumour formation.

Dlk1 has been implicated in the ovine callipyge (CLPG) phenotype characterized by a non-Mendelian mode of inheritance in which only heterozygous individuals inheriting the CLPG mutation from their father display muscular hypertrophy. Although attributed to a non-coding mutation in an intergenic region of the CLPG locus, the imprinting status is maintained in the locus but expression of the *DLK1, GTL2, PEG11*, and *MEG8* transcripts are augmented in *cis* [7]–[9]. By showing that Dlk1 protein is present in post-natal skeletal muscle of only callipyge animals, and by producing transgenic mice that recapitulate the CLPG phenotype by ectopically expressing the ovine C2 membrane-bound isoform of Dlk1, we have shown that Dlk1 is responsible, at least in part, for the manifestation of the CLPG phenotype [8]. These findings were corroborated by an additional study on the CLPG phenotype where the authors report expression of Dlk1 in a subpopulation of mononuclear cells co-expressing Pax7, a marker for resting and activated satellite cells [10]. In a recent study, we demonstrated that Dlk1 is expressed in rodent muscle during regeneration, in human fetal muscle and in myopathies, with localization to both satellite cells and myotubes [11].

Together, these data suggest that Dlk1 is likely to be involved in myogenesis/growth of skeletal muscle during normal embryonic development with expression possibly recapitulated in regenerating muscle. Despite these observations, the function of Dlk1 in muscle remains elusive. In other mesodermal tissues, such as adipose tissue and bone, Dlk1 acts as an inhibitor of terminal differentiation [12], [13]. Therefore, considering that maturation arrest is proposed as a factor in the development of rhabdomyosarcomas (RMS) [14] this study was designed to elucidate if skeletal muscle neoplasias express Dlk1, and further, to determine if Dlk1 in muscle influences myogenesis.

## Materials and Methods

### Ethics Statement

The use of human archival tissue for immunohistochemical analyses during this study was approved by The Regional Scientific Ethical Committee for Southern Denmark (20070075).

For isolation of primary myoblasts, biopsies were obtained from voluntary participants. The participants gave a written informed consent and The Regional Scientific Ethical Committee for Southern Denmark (S-20070079) approved the use of these biopsies for isolation of primary myoblasts and their subsequent propagation, analysis and use in the G_0_ model.

All animal experiments were performed in accordance with Danish Legislation on animal welfare and approved by the Danish Council for Supervision with Experimental Animals. Mice were housed under standard conditions, kept in a 12 hour light/dark cycle and had access to food and water ad libitum. The mice were provided with enrichment for improved care and to avoid stereotypic behavior. Mice showing any type of distress or illness were immediately euthanized by cervical dislocation. Following the surgical procedure (described below), the mice received a subcutaneous injection of Temgesic to alleviate any pain or discomfort by the procedure.

### Human Samples

All human tumor samples were obtained from the biobank at Department of Pathology, Odense University Hospital.

### Animal Model

Transgenic mice (TG) expressing the ovine C2 variant of *Dlk1* under control of the Myosin Light Chain Promoter (line D) and age-matched normal littermate controls (LC) (age 10–13 weeks) have been described previously [8].

### Animal Experiments

Dlk1 transgenic mice (TG) (n = 39) and littermate controls (LC) (n = 39) were anaesthetized using Avertine and regeneration was induced by a knife stab in m. gastrocnemius of both hind limbs. Mice were sacrificed by cervical dislocation at 0, 0.5, 1, 1.5, 2, 2.5, 3, 4, 5, 6, 7, 9 and 12 days post injury (n = 3 for each time point and phenotype). Both m. gastrocnemius were dissected out for each animal, one was fixed in 4% formalin and embedded in paraffin for histological analysis and the other stored in 2xNucleic Acid Purification Lysis Solution at −20°C (Applied Biosystems, Foster City, CA, USA) for RNA extraction and subsequent qPCR.

### Stable Transfection of C2C12 Cells with Mouse Full Length *Dlk1*

Stable transfection of *Dlk1* into the genome of C2C12 cells was performed using the Flp-In™ System (Lifetechnologies, Taastrup, Denmark).

First, a Flp-In™ C2C12 host cell line was established by inserting the plasmid pFRT™/lacZeo into the genome according to the manufacturer’s instructions using Lipofectamine2000™Reagent (Lifetechnologies). The plasmid was linearized using Sca1 (Lifetechnologies) prior to transfection. Clones were screened for number of integration sites by southern blotting as previously described [8] using a probe directed against the *LacZ* gene in the inserted fragments (data not shown). The transcriptional activity of the integrated sites was tested by β-galactosidase assay (data not shown), and the myogenic commitment of the cells was tested in a differentiation assay to ensure no loss of function due to the genomic integration (data not shown).

Full length murine Dlk1 was amplified from mouse pituitary gland cDNA [2] using PCR primers covering the 3′ and 5′ end of the mRNA. Fwd primer including a HindIII restriction site, a Ribosomal Binding Site and a start codon: 5′ ccccaagcttgagatgatcgccgaccggagc 3′. Rev primer including a Xho1 restriction site and a stop codon: 5′ ccccctcgagttagatctcctcatcaccagcct 3′. *Dlk1* cDNA and the vector pcDNA5™/FRT were digested with HindIII (Lifetechnologies) and XhoI (Lifetechnologies) at 37°C for 2 hours. *Dlk1* was inserted into the plasmid using T4 DNA Ligase (Lifetechnologies). The resulting Dlk1-pcDNA5™/FRT was co-transfected into the C2C12 host cell line with pOG44, expressing the FRT Integrase using Lipofectamine2000™Reagent according to the manufacturer’s instructions, followed by selection using 200 mg/ml Hygromycin and the resultant clones were screened for expression of Dlk1 using qPCR and immunocytochemistry (data not shown).

### C2C12 Cell Differentiation Assay

DLK1-C2C12 and C2C12 control cells were seeded (2000 cells/cm^2^) in 6-well plates (Nunclon™, VWR, Rødovre, Denmark), T25 flasks (Nunclon™, VWR, Rødovre, Denmark) or in LabTek©CC2 4-well chamber slides (Nunclon™) and grown in proliferation medium: DMEM/high glucose (Gibco, Lifetechnologies, Taastrup, Denmark), 10% FBS (Gibco) and 1% Penicillin/Streptomycin (P/S) (Gibco) until 100% confluence (day 3), where after the cells were changed to differentiation medium: DMEM/high glucose with 2% Horse Serum (Gibco) and 1% P/S. Cells were harvested after 1, 3, 5 and 8 days in culture. Cells from 6-well plates were harvested for RNA extraction and qPCR in 1xNucleic Acid Purification Lysis Solution (Applied Biosystems). T25 flasks were harvested for protein extraction and western blotting. Chamber slides were harvested for immunocytochemistry. Cells grown on coverslips or in T25 flasks were incubated +/−0.001 mg/ml Dlk1 antibody during differentiation (rabbit-anti-pref1, Millipore, AB3511).

### Culture of Rhabdomyosarcoma (RMS) Cell Lines

We used two different RMS cell lines, RD and A-204. Both are commercially available and were kindly provided by Camilla Frölich, University of Copenhagen. The cells were grown in growth medium, GM (DMEM w. 10% FBS, 1% penicillin and streptomycin (PS), Lifetechnologies) and differentiated for 7 days in DMEM containing 2%FBS, 1% penicillin and 25 pmol Insulin (Actrapid from Novo Nordic).

### Primary Human Myoblast Cultures

The primary human myoblast cultures were established from muscle biopsies taken from m. vastus lateralis as described previously [15].

### Isolation and Propagation of Human Myoblasts

Biopsies free of connective tissue were minced washed and dissociated with 0.05% trypsin-EDTA (Lifetechnologies) for 3×30 min. Harvested cells were pooled and Fetal Bovine Serum added as protease inhibitor. The isolated cells were seeded for up scaling (max 7 passages) on ECM-coated dishes (NUNC) after 15 min. of pre-plating and cultured in growth medium, GM (DMEM w. 10% FBS and 1% penicillin and streptomycin (PS)).

### G_0_ Arrest, Reactivation and Differentiation of Human Myoblast Cultures

The procedure for G_0_ arresting human myoblasts was modified from previous studies [16]. Proliferating human myoblasts were detached with 0.05% Trypsin-EDTA, pre-plated and transferred to suspension medium, SM, (DMEM with 2% methyl cellulose (Sigma-Aldrich), 10% FBS and 1% PS) and cultured in dishes with ultra low attachment surface (Corning) with a density of 150.000 cells/ml. This suspension culture condition makes the cells enter G_0_ arrest.

For harvesting cells from suspension medium the cells were washed twice in PBS. Cells were resuspended in lysis buffer (for RNA isolation) or plated on ECM-coated dishes and first cultured in growth medium followed by culture in differentiation medium, DM (DMEM with 2% FBS, 1% PS and 25 pmol Insulin (Actrapid from Novo Nordisk) for 5–7 days. Proliferating and differentiated cells were harvested in lysis buffer for RNA isolation. Pellets from all culture conditions were harvested for protein extraction.

### qPCR and RT-PCR

RNA was extracted from all tissue and cell samples using the ABI PRISM™ 6100 Nucleic Acid Prep Station (Applied Biosystems) according to the manufacturer instructions. cDNA synthesis was performed using the High Capacity cDNA Reverse Transcription Kit (Applied Biosystems).

#### Detection of endogenous Dlk1 splice variants in mouse muscles and in rhabdomyosarcomas (RMS cells)

Using primers spanning exon 5, all known splice variants of mouse Dlk1 could be detected since all splice sites are located within exon 5 [5]. This PCR reaction theoretically yields bands corresponding to the A (824 bp), B (671 bp), C/C_2_ (605/599 bp) and D/D_2_ (545/539 bp) forms of mouse Dlk1. Mouse Pituitary gland (PG) was used as positive control (20). Primer sequences: Fwd: 5′-TCTGCGAGGCTGACAATGTCTG-3′ Rev: 5′-CAGGATGGTGAAGCAGATGG-3′. The reaction was run at 57°C annealing temperature for 35 cycles using Taq Polymerase and 2M Betaine as additive.

Analysis of the endogenous splice variants was run on pools (containing 3 biological replicates each) of all time points from the Dlk1 TG/LC mouse injury study.

For detection of potential human splice variants in rhabdomyosarcomas we performed a PCR reaction on the two RMS cell lines, RD and A-204, using primers spanning exon 5 of the human Dlk1 sequence. Human placental and skeletal muscle tissue was used as positive control material and a no-template cDNA synthesis reaction was used as negative control. To verify the products obtained in the PCR reactions, the bands were extracted from the gel followed by sequencing (Eurofins MWG) and a BLAST search. Primer sequences: Fwd (located in exon 4): 5′-ACGGGCCCTGTGTGATCAACG-3′ Rev (located in exon 5): 5′-CCTCGTCGCCGGCCTCCTT -3′. The reaction was run at 60°C annealing temperature for 40 cycles using Taq Polymerase.

#### Global gene expression profile

qPCR reactions were run using TaqMan™ Low Density Array Cards (Applied Biosystems) in a set-up with 32 genes analyzed in triplicates of four samples simultaneously. Briefly, 400–500 ng cDNA in 100 µL were mixed with 100 µL TaqMan® 2x PCR MasterMix (Applied Biosystems) and the qPCR was run on the ABI PRISM™ 7900 HT Real Time PCR System (Applied Biosystems). Relative expression was analyzed using SDS2.1 (Applied Biosystems), GeNorm and qBase software [17], [18].

Briefly, raw data were extracted and analyzed in SDS2.1 using automatic detection of Ct-values, followed by export to qBase. Here the Ct-values were quality-checked, all triplicates were evaluated and a run was excluded if the difference in Ct within a triplicate >0.5. Various reference genes were analyzed: *Tbp*, *Pgk1*, *Hprt1*, *18s rRNA*, *Trfc, B2m, Gapdh, Gusb* and *Actb.* Before normalization of the data absolute values for all reference genes were exported to geNorm and the criteria for selection of reference genes used was a M-value<1.5 and a V<0.15. Based on these criteria *18s rRNA*, *Hprt1*, *Pgk1* and *Tbp* (mean CV%: 18.48 and mean M: 0.4422) were used for calculation of relative values in the mouse *in vivo* study, *Actb, Gusb* and *Pgk1* (mean CV%: 18.19 and mean M: 0.4557) were used for calculation of relative values from mouse *in vitro* cultures and PGK1, 18S, TBP, TFRC and B2M were selected as reference genes for human *in vitro* cultures.

#### Analysis of ectopic DLK1 expression in stably transfected C2C12 cells

Pre-designed TaqMan® Gene Expression Assays (Applied Biosystems) amplifying *Dlk1*, *Gapdh* and *18s rRNA* were run on 5 DLK1-C2C12 clones using an iCycler iQ (Bio-Rad Laboratories, Copenhagen, Denmark) according to the manufacturer’s instruction. All reactions were run in triplicates. *Gapdh* and *18s rRNA* were used to normalize gene expression levels. Relative expression was analyzed using iCycler (Bio-Rad) and Genex (Bio-Rad) software.

#### Real-Time single tube reactions

For analysis of genes not included in the TaqMan™ Low Density Array Card, TaqMan® Gene Expression Assays (Applied Biosystems) were used. The qPCR was run in triplicates on the ABI PRISM™ 7900 HT Real Time PCR System and analyzed using SDS2.1 (Applied Biosystems) and qBase. All mouse muscle sample reactions were run on *Decorin*, *TGFβ1* and *Follistatin*, in DLK1-C2C12 and C2C12 control cell lines reactions were run on *Decorin* and *Follistatin. Gapdh*, *18s rRNA, Tfrc* and *Pgk1* were used as endogenous control genes (mean CV% <20 and mean M<0.6).

### Histology, Immunohistochemistry (IHC) and Immunocytochemistry (ICC)

Formalin fixed and paraffin embedded mouse or human muscle tissue was cut in 4 µm sections and stained with sirius red (only mouse sections) and HE for morphological analysis. For IHC; Heat-Induced Antigen Retrieval was done by boiling the sections in Tris-EGTA buffer, pH 9.0 first 15 min at 900W then 9 min at 440W.The sections were allowed to cool in the buffer before they were blocked for endogenous peroxidase and biotin activity. The following primary antibodies were used: mouse-α-Pax7 1:10 (mouse sections) or 1∶200 (human sections) (Developmental Studies Hybridoma Bank, Iowa, USA), mouse-α-myogenin 1∶200 (mouse sections) or 1∶800 (human sections) (F5D, DAKO, Glostrup, Denmark), rat-α-mouse ki67 1:50 (Tec3, DAKO), mouse-α-p27 1:100 (Transduction Lab, Becton & Dickinson, Broendby, Denmark), rabbit-α-human FA1 1:200 (on mouse sections, detects ectopic ovine DLK1 expression [8]) or 1∶800 (on human samples) (private CHJ), rabbit-α-rat FA1 1:16000 overnight at 4°C (detects endogenous mouse DLK1), [19] (private CHJ), rabbit-α-chicken NCAM (Neural Cell Adhesion Molecule) 1∶2000 (AB5032, Chemicon International, Millipore, Copenhagen, Denmark), rabbit-α-myostatin (Abcam #996) 1∶100 and mouse-α-dystrophin 1∶10 (MAB1692, Chemicon International). For detection of mouse monoclonal antibodies, the ARK® kit (DAKO) was used for mouse sections, for human sections PowerVision (Poly-HRP anti-mouse, Novocastra, Leica Biosystems) was used. The Labelled Streptavidin-Biotin-System with rabbit-α-rat IgG 1∶200 (DAKO), the Envision+ HRP system (K4003, DAKO) or PowerVision (Poly-HRP anti-rabbit.Novocastra, Leica Biosystems) was used for polyclonal antibodies. Nuclei were counterstained using mayers haemalum w/4.5% chloralhydrate.

For ICC; cells were fixed 5 min in 4% formaldehyde, permeabilized in 0.5% Triton-X/TBS (only nuclei stainings) and blocked 10 min with 2% Bovine Serum Albumin/TBS before direct addition of primary antibodies: rabbit-α-rat FA1 1:2000, mouse-α-myogenin 1∶50, mouse-α-fast myosin (MY32) 1∶8000. Secondary antibodies employed were donkey-anti-mouse-488 (Alexa, Lifetechnologies) and goat-anti-rabbit-555 (Alexa, Lifetechnologies). Nuclei were detected with DAPI mounting medium (VECTASHIELD® Mounting Medium, Vector Laboratories, Inc., VWR, Rødovre, Denmark).

Both immunohistochemistry and immunofluorescence images were obtained with a Leica DM LB2 microscope and acquired using the digital camera Leica DFC 300F with the Leica FireCam software and composites of digitized images were assembled using Adobe Photoshop CS5 software.

### Morphometrics

The number of cells expressing Pax7, ki67, p27 and myogenin protein during regeneration in TG and LC mice was estimated on sections by counting positive nuclei (n = 3 for each phenotype at each time point). Morphometrics were performed using a Leica microscope equipped with a camera connected to a motorized cross board and a computer. By means of the CAST software (©2000 Olympus Denmark A/S), systematic fields for counting were selected to include the entire muscle section. The number of positive nuclei was compared to the total number of nuclei in the counted fields to give an estimate of the percentage of cells expressing the various proteins at a given time point investigated.

### Western Blotting

Total protein was extracted from the harvested pellets and samples were added loading buffer (Life Technologies) and sample reducing agent (Life Technologies), heated to 95° for 5 min and then loaded on a 4–12% Bis-Tris gel. The gel was run using MES buffer for 1 h at 150 V constant and transferred to a PVDF membrane (0.45 µm pore size) using electro-blotting for 1 h at 200 V constant, with stirring and cooling. The membrane was blocked for 1 h at room temperature with either 5% skimmed milk/TBS-T or 5% BSA/TBS-T and incubated with primary antibodies over night at 4°C, shaking (rabbit-anti-pref1, AB3511, Millipore) 1∶5000, rabbit-anti-myostatin (Abcam, #996, used for C2C12 cell analysis) 1∶500, rabbit-anti-beta actin (Cell Signaling Technology, #4967S) 1∶1000, rabbit-anti-myostatin (Abcam #98337, used for human myoblast analysis) 1∶250, goat-anti-hDlk1 (R&D systems, AF1144) 1∶1000. The following day blots were washed using TBS-T and incubated with goat-anti-rabbit-HRP (DAKO, p0448) 1∶2000 or rabbit-anti-goat-HRP 1∶1000 (DAKO, p0449) for 1 h at room temperature and developed using Enhanced Chemiluminescence kit (Lifetechnologies) and standard x-ray film.

### Statistics

Both in the qPCR analyses and the morphometrical analyses p-values were calculated for the comparison of transgenic mice with littermate control mice during the entire regeneration period and transfected cells versus controls for the entire time-period studied using two-way factorial ANOVA with an α-value of 0.05. P-values<0.05 were considered statistically significant. GraphPad Prism® 5 (GraphPad Software, Inc., San Diego, USA) were used for all analyses.

## Results

### Dlk1 is a Marker for Rhabdomyosarcomas and Rhabdomyomas

**Figure 1:**
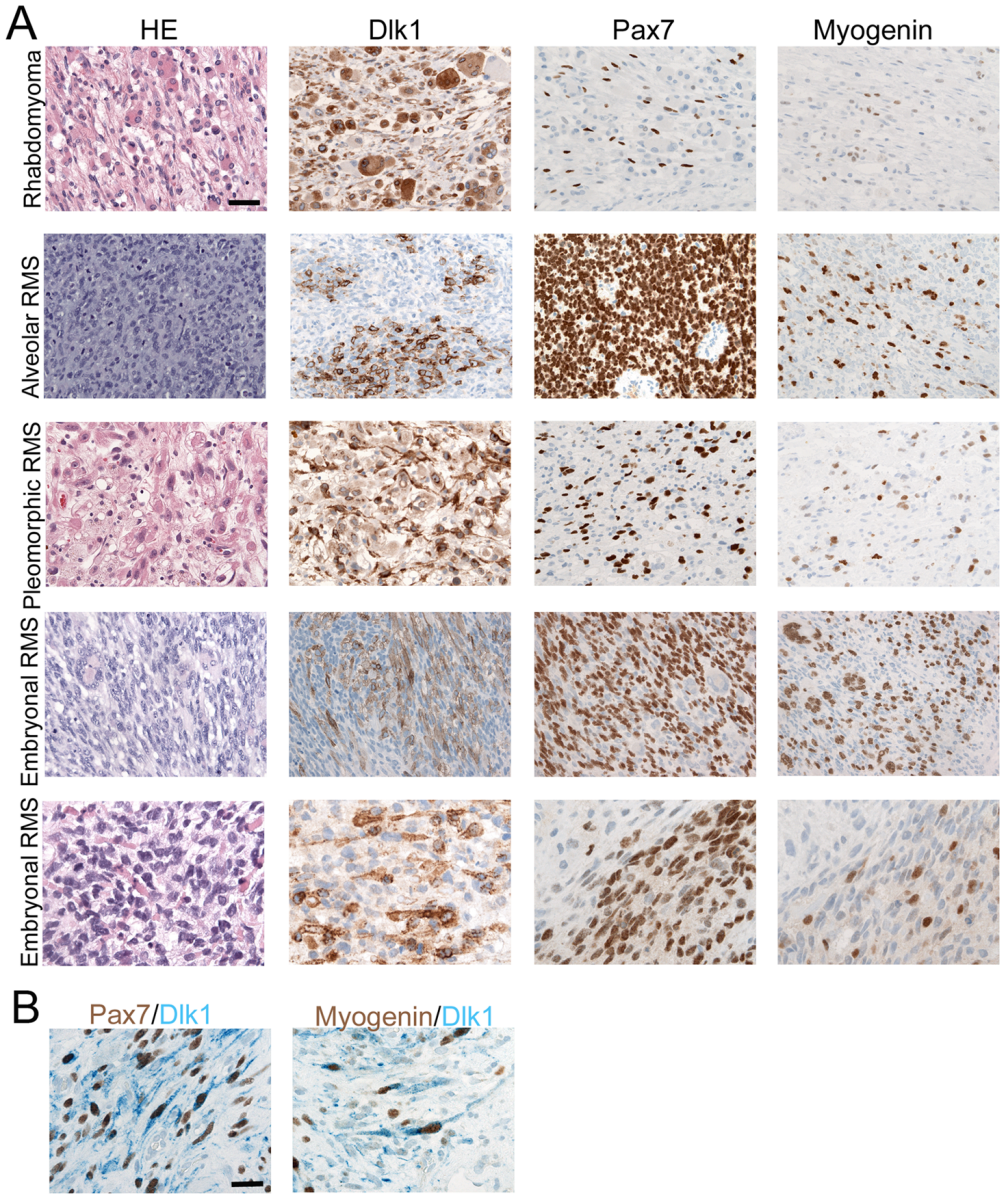
Immunohistochemical analysis of skeletal muscle tumors. A: Biopsies from rhabdomyoma and the 3 rhabdomyosarcoma subtypes, alveolar, pleomorphic and embryonal, were analyzed for expression of Dlk1, Pax7 and Myogenin. Dlk1 was strongly expressed by all skeletal muscle tumor types and can thus be considered a solid marker. Expression of Pax7 and Myogenin in all tumors confirmed the skeletal muscle origin. Scalebar representative for all images: 50 µm. B: Dlk1 expression was detected specifically in cells co-expressing either Pax7 or Myogenin, shown as double staining. Two examples of rhabdomyosarcomas with these co-expression patterns are shown. Scalebar representative for all images: 50 µm.

We first investigated Dlk1 expression in neoplastic lesions derived from skeletal muscle, adipose tissue, and bone. Eighteen of twenty-one skeletal muscle derived tumors representing embryonic, alveolar, and pleomorphic rhabdomyosarcomas as well as rhabdomyomas expressed Dlk1 (table 1, fig. 1). A single sarcoma of each subtype was negative. Compared to other myogenic markers, expression of Dlk1 showed similarity to Desmin and NCAM (Neural Cell Adhesion Molecule, CD56) in sensitivity and was strongly expressed compared to Pax7, Myogenin, and Actin.

**Table 1:** Immunohistochemical profile of rhabdomyosarcomas (RMS).

Type of RMS	Desmin	Pax7	Myogenin	NCAM	Dlk1
Embryonic	+++	+++	++	+++	+++
Embryonic	++	+++	+	++	N
Embryonic	+++	+++	+++	+++	++
Embryonic	+++	+++	++	+++	+
Embryonic	++	N	N	(+)	+
Embryonic	++	+++	+++	+++	+++
Embryonic	+++	N/A	+++	N/A	N/A
Embryonic	+++	+++	++	+++	++
Embryonic	++	++	+	+++	+++
Embryonic	+++	+++	++	+++	++
Embryonic	N/A	+++	++	+++	++
Alveolar	N	+	N	+++	N
Alveolar	+	N	N	+	+++
Alveolar	+++	+++	++	+++	++
Alveolar	+++	N/A	N/A	+++	N/A
Pleomorphic	+++	+++	++	+++	++
Pleomorphic	++	+	++	+++	+
Pleomorphic	++	N	+	+++	+
Pleomorphic	+++	(+)	+	+++	N
Pleomorphic	+++	N	N	N	+
Rhabdomyoma	+++	++	++	+++	+++
Rhabdomyoma	+++	++	N	+++	+++
Rhabdomyoma	+++	+++	(+)	+++	++

N = negative, N/A = not available.

**Figure 2:**
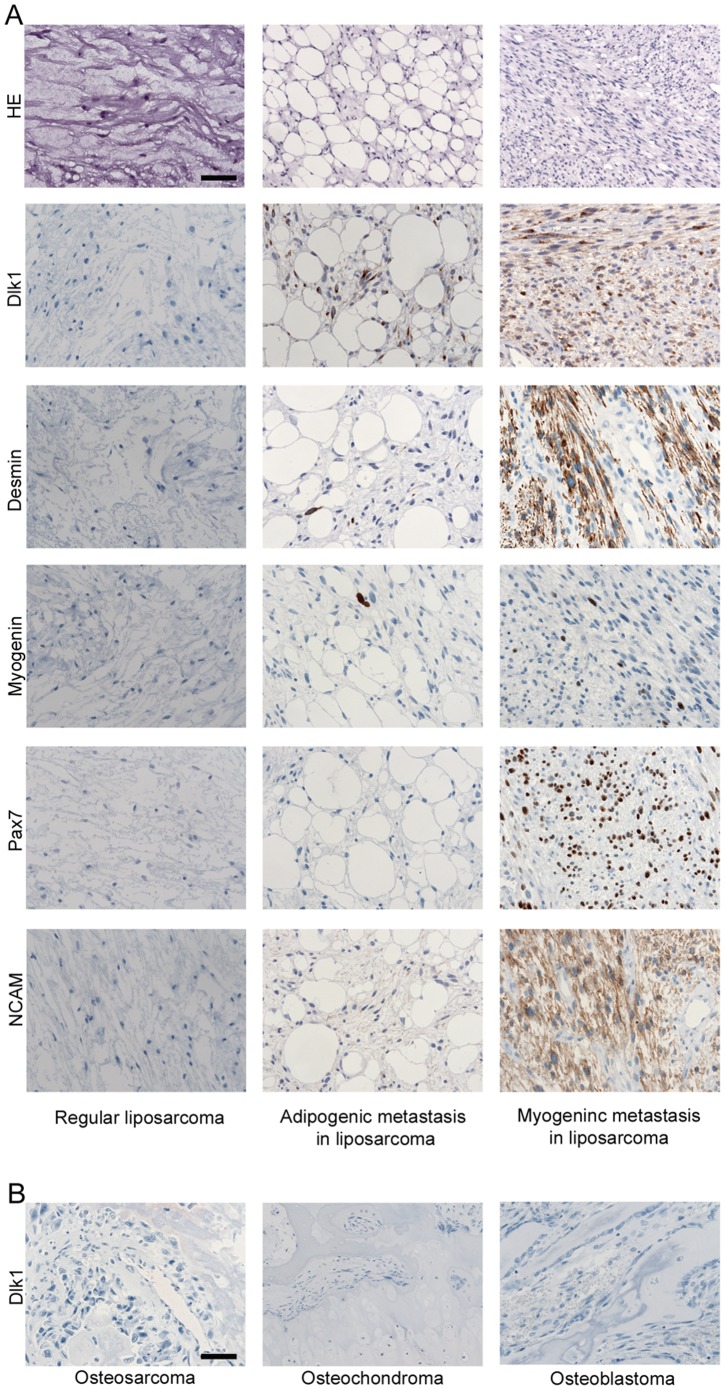
Immunohistochemical analysis of tumors with adipogenic or osteogenic origin. A: A biopsy from a liposarcoma with an adipogenic and a myogenic metastasis was investigated for expression of Dlk1, Desmin, Myogenin, Pax7 and NCAM. All myogenic markers were predominantly detected in the myogenic metastasis and not in the main liposarcoma or the adipogenic metastasis. Accordingly, Dlk1 was primarily expressed in the myogenic metastasis. Few Dlk1 positive cells could, however, be detected in the adipogenic metastasis, where few cells were found to express Desmin and Myogenin as well. This supports the observation that Dlk1 is a strong marker for tumors with skeletal muscle phenotype. Scalebar representative for all images: 50 µm. B: Biopsies from tumors with osteogenic origin were analyzed for expression of Dlk1 and all types analyzed (osteosarcoma, osteochondroma and osteoblastoma), were negative for Dlk1 protein expression. Scalebar representative for all images: 50 µm.

In liposarcomas (adipose tissue tumors) (table 2) five out of nineteen tumors expressed Dlk1. Of these, three co-expressed desmin and one co-expressed NCAM. None of nine bone-derived tumors expressed Dlk1 (table 3). In one of the liposarcomas, intra-abdominal metastases developed. Part of these metastases bore resemblance to a liposarcoma, and the others showed a more cellular pattern, predominated by spindle-shaped cells. Dlk1 was detected in both parts of the metastases, but the expression was most prominent in the latter. The expression pattern of Dlk1 in the two parts correlated well with expression of the myogenic markers Desmin and NCAM; the liposarcoma-like part showed some Desmin and NCAM and a few Myogenin positive nuclei, while the more cellular part presented with a strong Desmin, NCAM, Myogenin and Pax7 staining (fig. 2). This suggested that Dlk1 expression in liposarcomas could be indicative of a myogenic capacity.

**Table 2:** Immunohistochemical profile of liposarcomas.

Diagnosis	Desmin	Pax7	Myogenin	NCAM	Dlk1
Well differentiated	N	N	N	N	N
Well differentiated	N	N	N	N	N
Well differentiated¤	+	N	N	N	+
Well differentiated¤¤	+	N	+	+	+
Well differentiated	N	N	N	N	N
Well differentiated	N	N	N	N	N
Dedifferentiated	N	N	N	N	N
Dedifferentiated (see fig. 2)	+++	++	++	++	+++
Myxoid	N	N	N	N	N
Myxoid	N	N	N	N	N
Myxoid/round cell	N	N	N	N	++
Myxoid/round cell	N	N	N	N	N
Myxoid/round cell	N	N	N	+	N
Pleomorphic	+	N	N	++	N
Pleomorphic	N	N	N	N	N
Pleomorphic	N	N	N	+++	N
Mixed type¤¤¤	N	N	N	+	+
Mixed type	+++	N	N	N	N

¤ ) Desmin and Dlk1 were present in the same cell population.

¤¤ ) In a single location, could be derived from infiltrated muscle.

¤¤¤ ) Dlk1 positive cells correspond with NCAM positive areas.

**Table 3:** Dlk1 expression in osteogenic lesions.

Diagnosis	Dlk1
Osteogentic sarcoma	N
Osteogentic sarcoma	N
Osteogentic sarcoma¤	N
Osteogentic sarcoma	N
Osteoblastoma	N
Osteochondroma	N
Osteochondroma	N
Osteochondroma	N

¤ After chemo therapy.

Thus in neoplasias derived from mesenchymal tissues, rhabdomyosarcomas appear to be the only tumor consistently expressing Dlk1, and expression is independent of subtype.

### Localization of Dlk1 in Rhabdomyomas and Rhabdomyosarcomas

In all Dlk1-positive skeletal muscle tumors under investigation expression was localized to multinuclear cells and/or mononuclear cells (fig. 1). Dlk1 positive cells were detected with co-expression of Pax7 and with co-expression of Myogenin. Thus, Dlk1 expression was associated both with early activation of satellite cells (Pax7) and also with late myogenesis (Myogenin), markers suggesting that Dlk1 has a broad window of activity.

### Transgenic Expression of Ovine C2-Dlk1 in Mouse Skeletal Muscle Accelerates Myotube Formation during Early Regeneration

**Figure 3:**
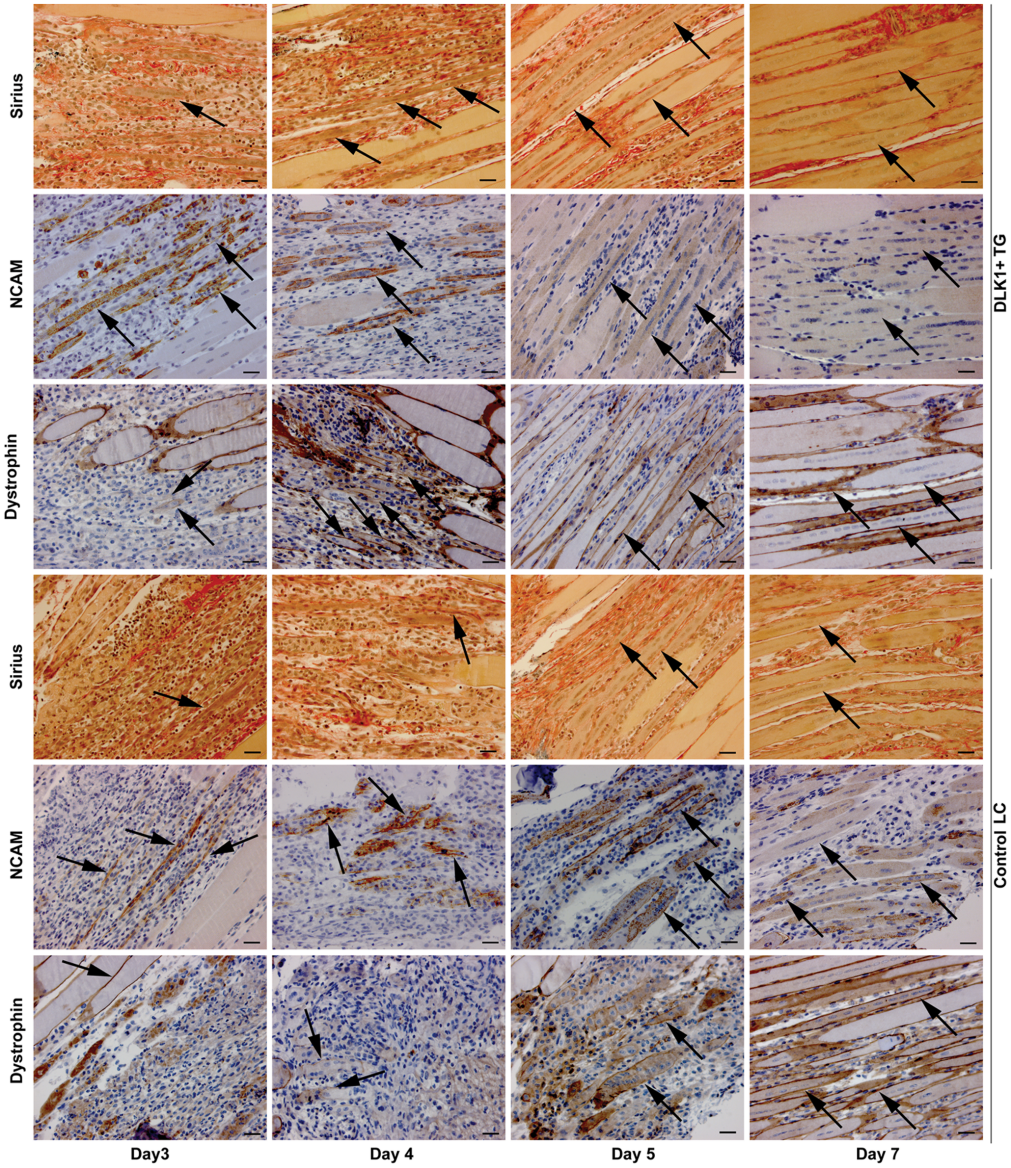
Dlk1 transgenic mice display an initially enhanced formation of myotubes following injury. A morphological analysis of skeletal muscle regeneration in Dlk1 transgenic mice (TG) and littermate control mice (LC) after knife stab injury was performed. Days 3, 4, 5, and 7 post injury is represented for each genotype in a sirius, NCAM (Neural Cell Adhesion Molecule) and dystrophin staining. Dlk1 TG mice display an early presence of newly formed myotubes at day 3 of which most express NCAM and few express dystrophin (arrows). In comparison, the LC control mice only have few myotubes at day 3 as indicated by sirius and NCAM (arrows), and the myotubes have no dystrophin in the membranes until day 5 (arrows). The regenerative response remains accelerated at days 4 and 5 in the Dlk1 TG mice compared to the controls (arrows). Even though the LC control mice display structured muscle tissue at day 5 with expression of both NCAM and dystrophin, the muscle architecture continues to appear more mature in the Dlk1 TG mice at this time point. Scalebar: 30 µm.

Considering that Dlk1 appears to be consistently associated with the muscle-derived neoplasias combined with our previous demonstration of expression in human, fetal muscle and in myopathies [11], we next wondered how Dlk1 influences immature myogenic cells and myogenesis. In our study we employed 2 models. The first model was a transgenic mouse with over expression of ovine membrane-bound Dlk1 isoform C2 in type 2 myofibers driven by the myosin light chain promoter [8] and the other model was an in vitro study with over expression of mouse full length, soluble isoform A in C2C12 myoblasts. In the C2 isoform model we performed a knife stab injury in m. gastrocnemius of transgenic mice (TG) and in wild-type littermate controls (LC). Morphologically, the sequence of repair events was the same in both groups of mice as studied by H&E (not shown) and Sirius Red staining (fig. 3). However, at day 3 the Dlk1 TG mice presented substantially more myotubes in comparison to the LC mice. The accelerated formation and maturation of myotubes continued through days 4–5, but from day 6 the regeneration occurred similarly in Dlk1 TGs and LCs. Immunohistochemical staining for NCAM and Dystrophin supported the morphological observations (fig. 3).

**Figure 4:**
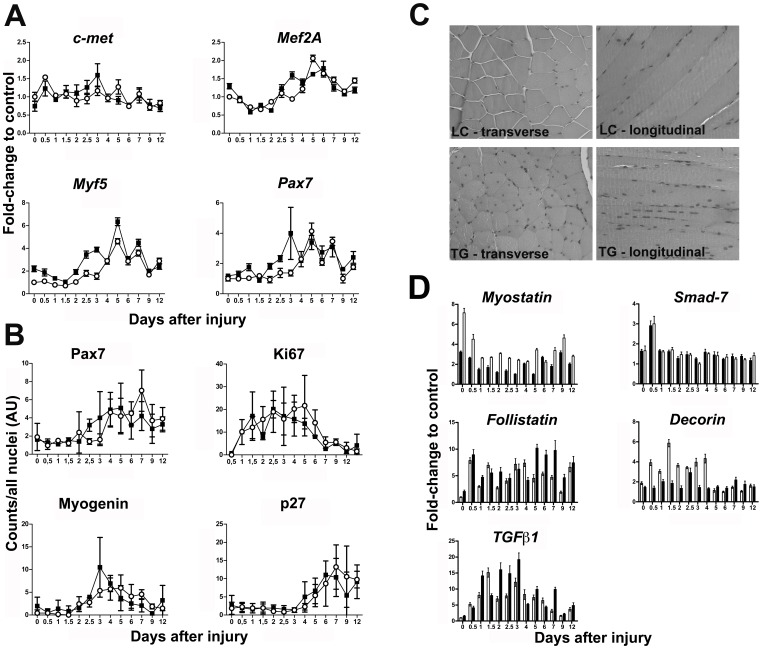
Analysis of skeletal muscle regeneration and the myostatin regulatory pathway in Dlk1 transgenic (TG) and littermate control (LC) mice by qPCR and morphometrics. A: qPCR analysis of *c-met*, *Mef2a, Myf5*, and *Pax7*, *Myod* mRNA levels, during regeneration of Dlk1+ TG and LC control mice. The relative expression levels were normalized to expression of the reference genes *18s rRNA*, *Hprt1*, *Pgk1* and *Tbp* and the mRNA levels were calculated as fold-change to uninjured LC control muscle (calibrator = 1) for both genotypes. The qPCR represents a pool of *m. gastrocnemius* harvested from 3 mice for each time point and genotype and was run in triplicates. Black squares: Dlk1 TG mice +/− SD. Open circles: LC control mice +/− SD. B: Morphometric analysis of Pax7, ki67, myogenin and p27 protein expression during regeneration of Dlk1 TG and LC control mice (n = 3 in each group for each genotype). Black squares: Dlk1 TG mice +/− SD. Open circles: LC control mice +/− SD. C: Presence of centrally localized nuclei in Dlk1 TG muscle compared to LC controls is shown both as transverse and longitudinal sections representing 2 different individuals for each genotype. D: qPCR analysis of *Myostatin, Follistatin, TGFβ1, Smad7* and *Decorin* mRNA expression during regeneration of Dlk1 transgenic mice and littermate controls. The qPCR is presented as fold-change to uninjured LC control muscle (calibrator = 1). The relative levels have been determined by normalizing the data to *18s rRNA*, *Gapdh*, *Hprt1*, *Pgk1* and *Tbp* as described in materials and methods. Black bars: Dlk1 TG mice +/− SD. White bars: LC control mice +/− SD.

We investigated further this initial difference between TG and LC mice by Real-Time PCR. We focused our analysis on expression of the satellite cell and myogenic regulators *C-met, Mef2a, Myf5 and Pax7* (for review, see [20]). Gene expression was analyzed as fold-change using uninjured LC muscle (day 0, expression equal to 1) as calibrator in both Dlk1 TG and LC muscle to allow for detection of initial changes induced by the Dlk1 transgene even in uninjured muscle. We observed that the early myogenic marker *Myf5* was differentially expressed between the two genotypes with a generally higher expression in the Dlk1 TG mice (fig. 4A, p<0.05). This elevation was observed from day 0, which supported the initial observed enhancement of regeneration.

The other markers analyzed using qPCR as well as a morphometric analysis of immunohistochemically stained sections for Pax7, Myogenin, p27 and Ki67 trended similarly to *Myf5*, but did not reach statistical significance (fig. 4A, B).

Despite the near-normal gene expression levels of myogenic factors during regeneration we observed that in contrast to their littermate control, the Dlk1 transgenic mice presented with centrally located nuclei in their myofibers suggesting an immature state of their skeletal muscles [21] (fig. 4C).

### Transgenic Ovine Dlk1-C2 Down Regulates Endogenous Dlk1 Protein Expression during Regeneration

**Figure 5:**
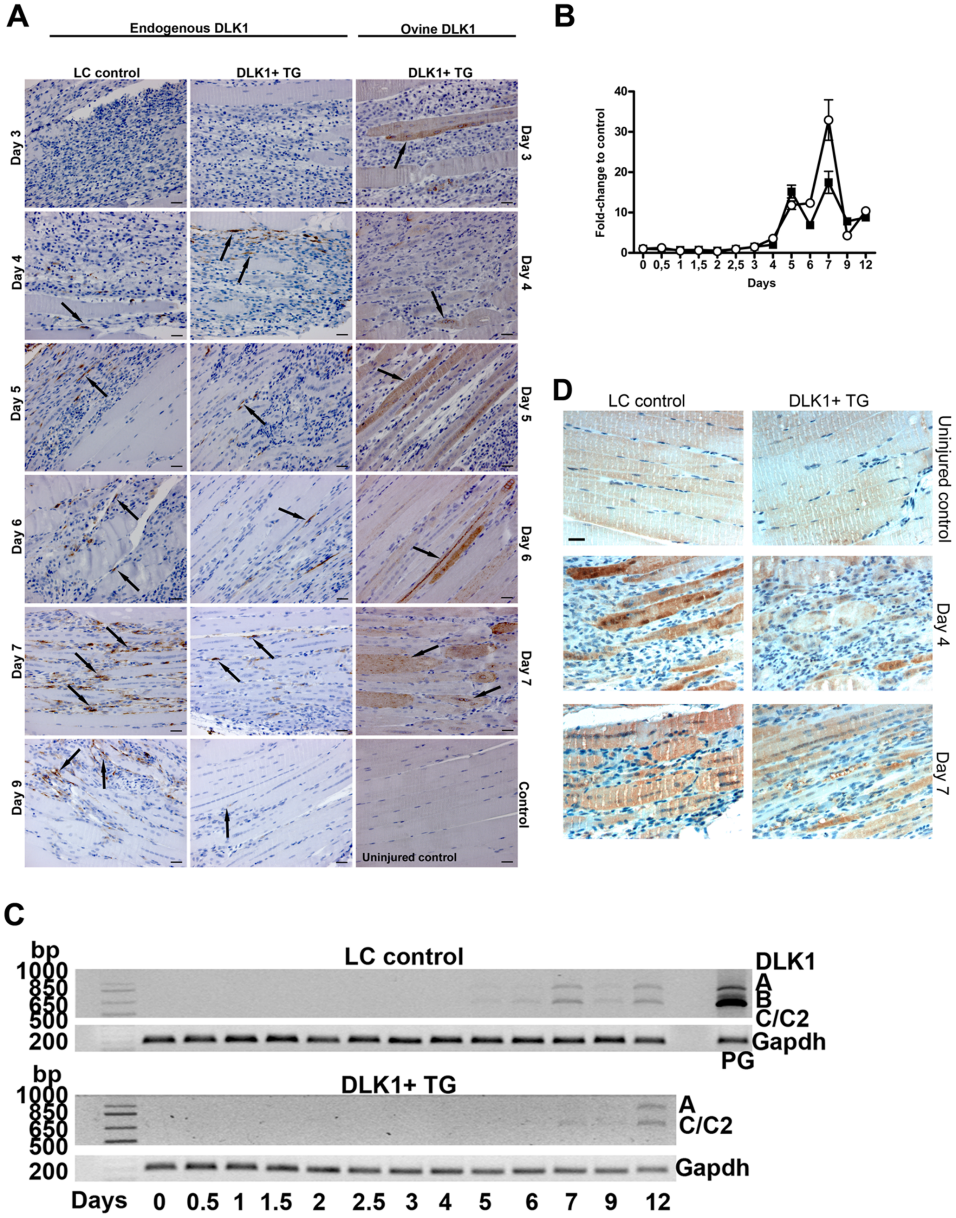
Ectopic ovine Dlk1 inhibits expression of endogenous Dlk1 and affects myostatin expression pattern. A. Endogenous Dlk1 expression was detected during regeneration using rabbit-anti-murine FA1 antibody and days 3–9 following stab wound are represented. Anti-human FA1 antibody cross-reacts with the ovine form of Dlk1/FA1 [8]. Days 3–7 following stab wound are represented. A staining of uninjured LC control muscle confirms that the staining of the ovine form is specific. Scalebar: 50 µm. In LC control mice endogenous Dlk1 is expressed in mononuclear cells located within the injury and primarily observed along the periphery of newly formed myotubes (arrows). In Dlk1 TG mice the expression of Dlk1 is strongly inhibited within the muscle injury and only few mononuclear cells expressing Dlk1 can be observed (arrows). Ectopic ovine Dlk1 is expressed only in the TG mice and not by LC mice. The expression is under control by the myosin light chain promoter and is primarily expressed by myotubes and mature muscle fibers (arrows). B. qPCR analysis of endogenous *Dlk1* mRNA confirms the down regulation by ectopic ovine Dlk1. Dlk1 was included in the TaqMan™ Low Density Array Card set-up and calculated as described in materials and methods. Black squares: Dlk1 TG mice +/− SD. Open circles: LC control mice. C. RT-PCR analyses of the Dlk1 splice variants present during regeneration. The primers used span exon 5 which contain all splice sites of Dlk1 and theoretically yields bands corresponding to the A (824 bp), B (671 bp), C/C_2_ (605/599 bp) and D/D_2_ (545/539 bp) forms. Pituitary gland (PG) is used as positive control. This analysis shows that the splice variants present are full-length A, C/C_2_ and confirms the down regulation of endogenous Dlk1 by ectopic Dlk, with all splice variants being equally affected. D. Immunohistochemical analysis of myostatin expression in Dlk1 TG and LC mice. Scalebar representative for all images: 20 µm. Myostatin expression is generally patchy and variable both in TG and LC mice, but TG mice present a less intense staining.

Mononuclear cells in littermate control mice express endogenous Dlk1 during regeneration. Expression starts at days 4–5 when differentiation of myoblasts, fusion and fiber maturation are the predominant physiological events, peaking at day 7 and declining again as the muscles mature (fig. 5, arrows indicate Dlk1 positive staining). In the TG group, the presence of ovine Dlk1 (fig. 5) reduced the expression of endogenous Dlk1 (fig. 5, black arrows). One possible explanation might be that ovine Dlk1 is detected by endogenous DLK1 (auto) regulatory mechanisms. The down regulation of endogenous DLK1 by ectopic Dlk1 was also detected by qPCR (fig. 5). Further analysis of the different murine *Dlk1* splice-variants (fig. 5) revealed that the dominant forms were the full length A and the C/C_2_ variants in both genotypes. In addition, the RT-PCR demonstrated further a down regulation of both variants in the TG mice. This supports the existence of a regulatory mechanism by which endogenous Dlk1-A and -C/C2 levels are modulated by transgenic, ovine Dlk1-C2.

### The Myostatin Pathway is Affected by Transgenic Ovine Dlk1

Myostatin, a member of the TGFβ superfamily, is a known inhibitor of muscle growth active in late regeneration [22], [23]. We observed that *myostatin* mRNA levels were significantly lower in the Dlk1 TG mice compared to controls in both uninjured muscles and also during regeneration (fig. 4C, p<0.001). Using immunohistochemical staining we detected a patchy expression of myostatin protein in both genotypes, with expression in TG mice being less pronounced. Reduced myostatin protein expression was detected throughout regeneration and thus corroborated the qPCR result (fig. 5D). The reduction in, but not absence of, myostatin in TG mice is in accordance with the mild muscular hypertrophy of these mice [24].

Prompted by the observed effect on myostatin expression levels we analyzed the mRNA expression of *TGFβ1*, *Smad7, Decorin* and *Follistatin*, all factors involved in regulation of the myostatin signaling pathway [25]–[27]. *Decorin* was downregulated in TG mice compared to controls (fig. 4, p<0.05), whereas *Follistatin* showed a general upregulation in expression from day 5–12 in TG mice (fig. 4, p = 0.05). No difference between genotypes was observed for *TGFβ1* and *Smad7* (*TGFβ1:* fig. 4, p>0.05; *Smad7*: fig. 4, p>0.05).

These results support the observations that Dlk1 interacts with the myostatin-signaling pathway in skeletal muscle. However, this effect does not appear to be mediated through changes in mRNA expression of the inhibitory Smad7 or the ligand TGFβ1.

### A-Dlk1 Inhibits Mouse Myoblast Differentiation in vitro

**Figure 6:**
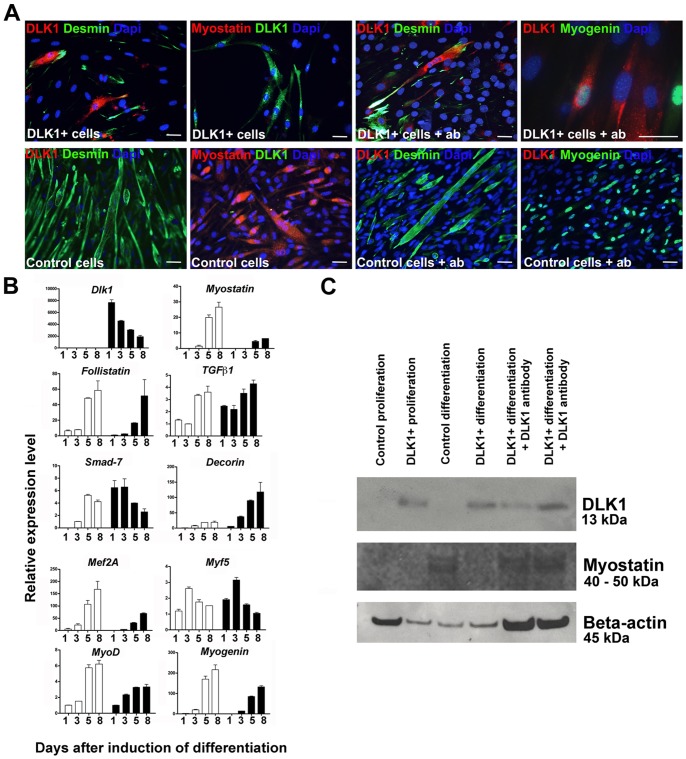
Dlk1 inhibits myoblast differentiation *in vitro*. A: C2C12 cells were constructed to constitutively express full-length Dlk1 under control by the CMV promotor, DLK1-C2C12. Cell morphology and myogenic differentiation potential was analyzed by immunocytochemistry. Forced expression of Dlk1 resulted in inhibition of myogenic differentiation observed as no formation of myotubes, lack of myostatin expression and reduced Desmin staining compared to C2C12 parental control cells. Addition of Dlk1 antibody to the culture supernatant during differentiation reverted the effect of forced Dlk1 expression observed as a regained ability to form myotubes and express myogenin compared to control cells. Scalebars: 20 µm in all images. B: DLK1-C2C12 cells (DLK1) and C2C12 parental control cells were cultured under proliferating conditions for 3 days followed by differentiation for another 5 days. Cells were harvested and analyzed at 1, 3, 5, and 8 days in culture. The differentiation process was analyzed by qPCR of *Dlk1*, the myogenic markers *Mef2a*, *Myf5*, *Myod*, and *Myogenin* in addition to *Myostatin*, *Follistatin*, *TGFβ1*, *Smad7* and *Decorin*. All qPCR analyses were run in triplicates on duplicate analyses containing triplicate samples (n = 2 for each time point) and presented as relative expression levels with normalization to.*Actb*, *Gusb*, *Pgk1*, *Gapdh* and *Tfrc* as described in materials and methods. Black bars: DLK1^+^ cells; white bars: control cells. C: Dlk1 (13 kDa band) and myostatin (a double band corresponding to 40–50 kDa) protein level was analyzed with western blotting during proliferation and differentiation of DLK1-C2C12 and control cells. Dlk1 protein was only expressed by DLK1-C2C12 cells and not by control cells. Myostatin protein was expressed in control cells during differentiation but not during proliferation of either of the cells lines or during differentiation of DLK1-C2C12 cells. However, addition of Dlk1 antibody resulted in expression of myostatin by the DLK1-C2C12 cells during differentiation. Beta-actin (45 kDa) was used as control.

The mouse model of Dlk1 over expression was constructed to imitate the ovine callipyge phenotype, expressing ovine Dlk1-C2 in myofibers only [8]. To study directly the effect of over expression of Dlk1 in the mononuclear, myogenic precursor cells, full length murine *Dlk1* was stably transfected into the genome of C2C12 cells (DLK1-C2C12), which do not express Dlk1 in their wild type state (fig. 6B).

Morphologically DLK1-C2C12 cells did not differ from the C2C12 controls. However, the DLK1-C2C12 cells were unable to differentiate as evidenced by a lack of myogenin-positive nuclei and of myotube formation (fig. 6A). In accordance with the observations from the in vivo regeneration study, we noticed a down regulation of *myostatin* mRNA and protein in DLK1-C2C12 cells (fig. 6B, C, p<0.01). Furthermore, the myogenic markers *Mef2a* (fig. 4B, p<0.05), *Myod* (fig. 6B, p<0.05) and *Myogenin* (fig. 6B, p<0.05) were all expressed at lower levels in DLK1-C2C12 cells during differentiation while *Myf5* mRNA levels were expressed at a higher level days 1–3 in DLK1-C2C12 cells (fig. 6B, p<0.05).

This prompted additional analysis of genes involved in the myostatin signaling pathway. *Smad7* (fig. 6B, p<0.05) was expressed at a higher level at days 1–3 followed by a decrease in expression compared to that of the control cells around days 5–8. *Tgfβ1* (fig. 6B, p>0.05) showed no difference. A generalized up regulation of *Decorin* mRNA levels was observed in DLK1-C2C12 from day 3 to day 8 during differentiation (fig. 6B, p<0.05), while no differences in *Follistatin* expression were observed (fig. 6B, p>0.05). ICC and WB analysis for myostatin likewise showed inhibition of protein expression (fig. 6A, C).

Thus, ectopic expression of Dlk1 isoform A in myoblasts modulated the expression of myogenic regulators and inhibited differentiation into myotubes.

### Blocking Antibodies Rescue the Differentiation Process in DLK1-C2C12 Myoblasts

We posited that Dlk1 could have a direct internal effect or an autocrine/paracrine effect based on secretion of the soluble form. Application of blocking antibodies to the DLK1-C2C12 myoblasts rescued the capability of the cells to initiate differentiation observed as myotube formation and Myogenin expression (fig. 6A). The application of antibodies also normalized the myostatin levels (fig. 6C). This indicates an autocrine/paracrine inhibitory effect of the secreted but not the internal Dlk1.

### Dlk1 and Myostatin are Reciprocally Expressed in Primary Isolated Human Myoblasts

**Figure 7:**
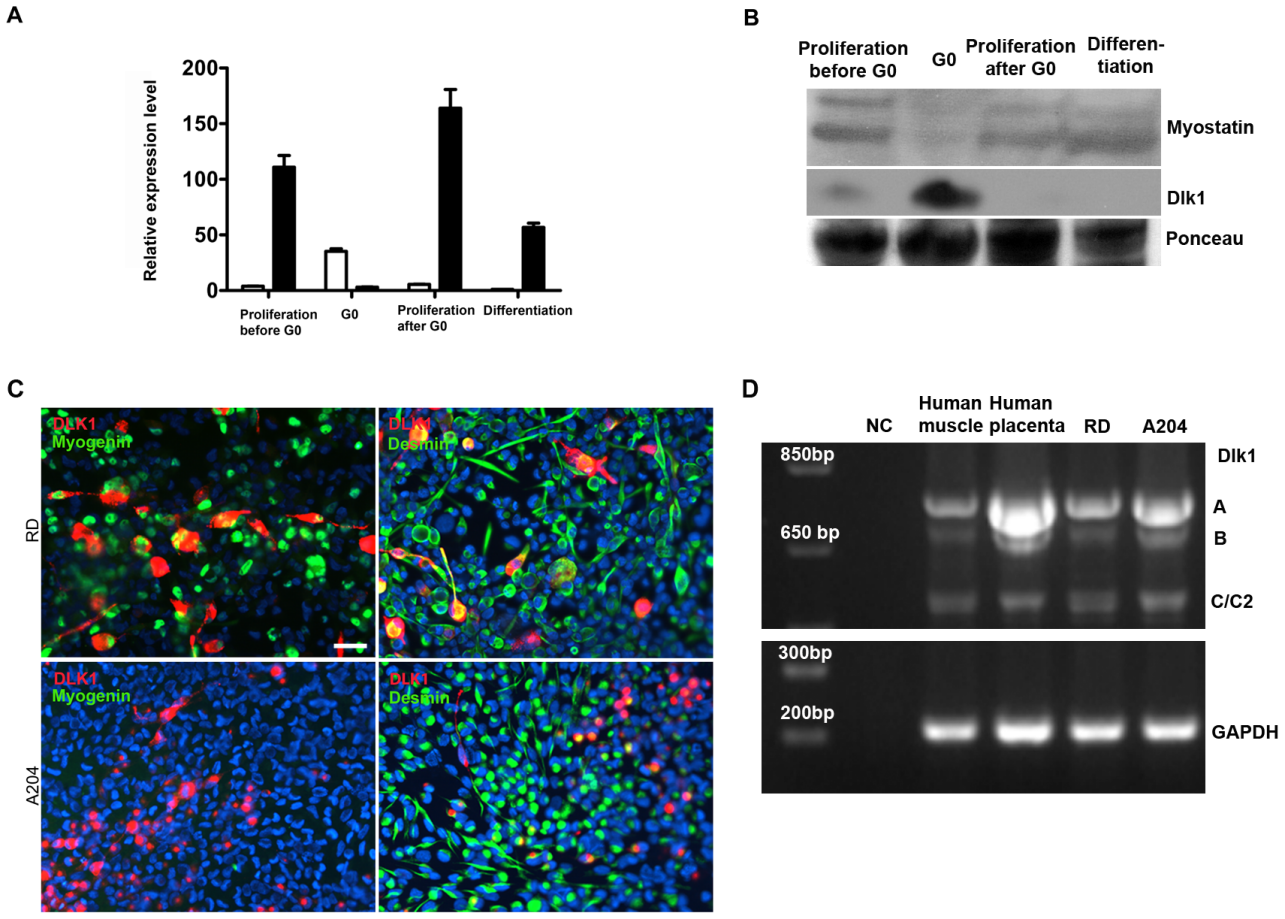
Dlk1 and myostatin display an inverse relationship in human myoblasts, and the expression of Dlk1 is observed in human rhabdomyosarcoma cell lines. A: Expression of Dlk1 and myostatin in two human myoblast cultures during G_0_ arrest, proliferation before and after G_0_, and differentiation. We found that myostatin was highly upregulated during proliferation and differentiation while Dlk1 was downregulated, thus an inverse relation was observed. B. Dlk1 (app. 30 kDa product) and myostatin (a double band around 26 kDa, corresponding to the cleavage product) protein level was analyzed with western blotting during proliferation before and after G_0_, during G_0_ (cell cycle arrest) and differentiation of primary isolated human muscle cells. Myostatin protein was expressed during proliferation and differentiation but down regulated during G_0_. Dlk1 protein was predominantly expressed by G_0_ arrested myoblasts. Ponceau Red staining (a common 70 kDa band is shown) confirmed equal loading. C: The expression of Dlk1, Myogenin and Desmin was studied in two human rhabodomyosarcoma cell lines, RD and A204 after induction in differentiation medium. We found that both cultures were Dlk1 and Desmin positive, while only RD cells were Myogenin positive. None of the cultures were able to form myotubes when cultured in differentiation medium. Scalebar representative for all images: 20 µm. D. RT-PCR analysis of Dlk1 splice variants present in RMS cells. The primers used span exon 5 and should thus provide bands for all possible splice variants present. The analysis shows the presence of 3 bands in both RMS cell lines and in the positive control tissue used, placenta and human muscle. These bands correspond to the full length A, and two smaller products most likely the B and C/C2 variants. All bands were sequenced and verified to be Dlk1.

If Dlk1 and myostatin expression in human muscle are inversely regulated as observed in mouse muscle and mouse myoblasts, a reciprocal expression should be displayed in human myoblast cultures as well. To explore this possibility, primary isolated human myoblast cultures were analyzed during proliferation, G_0_ arrest, proliferation after G_0_, and differentiation (fig. 7A). We observed that during proliferation, both before and after G_0_, as well as during differentiation, myostatin mRNA levels were highly up regulated compared to Dlk1 mRNA expression (fig. 7A). Notably, during G_0_ Dlk1 mRNA expression was highly up regulated and myostatin mRNA level was down regulated. The reciprocal expression was corroborated on protein level by western blotting (fig. 7B). These results demonstrate clearly that an inverse relationship between Dlk1 and myostatin exists, and furthermore, Dlk1 could be involved in controlling the quiescent state (G_0_) of human satellite cells.

### Dlk1 is Present in Human Rhabdomyosarcoma Cell Lines

We next analyzed two sarcoma cell lines, RD and A-204, for muscle marker expression to elucidate any similarity to myoblasts. During proliferation and after induction of differentiation (growth in low-serum medium) RD cells expressed Desmin and Myogenin (fig. 7 C), but we also detected Myosin heavy chain fast (MHCf), MyoD and NCAM (data not shown), while A-204 only expressed Desmin but not Myogenin (fig. 7C). Growth in low-serum medium failed to induce fusion and myotube formation in both cell lines (fig. 7C). Both cultures were Dlk1-positive when grown in low-serum medium (fig. 7C), while only A-204 was Dlk1-positive during proliferation (data not shown).

Dlk1 was detected in both rhabdomyosarcomas and RMS cell lines, thus it was of interest to analyze the tumor cells for specific Dlk1 splice variants. Using RT-PCR and primers spanning exon five, we detected bands corresponding to the full length A, the B and the C/C2 splice variants in placenta, muscle, and both RMS cell lines. All bands were verified by sequencing (fig. 7D). This suggests that no specific Dlk1 isoform can be attributed to RMS tumor progression.

Based on the results from mouse myoblast cultures, where the inhibitory effect of Dlk1 could be reversed using blocking antibodies, we tested if the addition of Dlk1 antibody to the sarcoma cell lines could induce myotube formation, since this could be a target for intervention in treatment of these tumors. However, the addition of Dlk1 antibodies to the culture medium did not induce myotube formation (data not shown), suggesting that the mechanism in the skeletal muscle tumors is more complex.

## Discussion

### Dlk1 is a Marker for Skeletal Muscle-derived Tumors

Rhabdomyosarcoma (RMS) is the most common childhood soft tissue sarcoma [28]. All three subtypes express muscle specific proteins notably Pax7 and Pax3 and the myogenic regulatory factors (MRFs) [29]. In myogenic cells these factors are responsible for commitment to the skeletal muscle lineage and control of myogenic differentiation [20]. Despite expression of these factors in RMS they fail to complete differentiation, which could be a central element in RMS biology.

Here, we demonstrate that Dlk1 was widely expressed in RMS. However, in mesenchymal tumors derived from other tissues, expression was low or undetected. In liposarcomas Dlk1 expression was paralleled by expression of other characteristic muscle markers. It has been shown previously that liposarcomas can display focal rhabdomyosarcoma differentiation [30], as confirmed by our studies (fig. 2). Interestingly, it has been shown that activation of sonic hedgehog signaling in adipocytes can give rise directly to skeletal muscle tumors with resemblance to embryonic RMS [31]. However, the consistent finding of Dlk1 in combination with muscle markers even in the liposarcomas suggests that Dlk1 is a direct element of the RMS characteristic regardless of cellular origin.

We observed Dlk1 co-localization with both Pax7 and Myogenin corresponding to the Dlk1 expression pattern in both human fetal and pathologic muscle [11], suggesting that Dlk1 could influence both proliferation and differentiation of RMS. White et al. [10] support this dual effect; they suggest that Dlk1 is involved in control of fetal myoblasts as well as myofiber-hypertrophy. The observation that expression of Dlk1 could be a hallmark for RMS is also indicated by observations of gene expression in childhood tumors [6]. Thus Dlk1 could be a highly sensitive and specific marker for RMS.

### Dlk1 and Myogenesis

Transgenic ovine Dlk1-C2 was shown *in vivo* to down regulate endogenous Dlk1 suggesting an auto regulatory feedback mechanism. Both reduction in the amount of endogenous protein and reduction in Dlk1 positive cells during regeneration were observed indicating a trans cellular/non cell autonomous effect of the membrane bound ovine C2 isoform. The observed downstream effects were a reduction in myostatin expression with only minor effects on the MRFs observed as a slight enhancement of the myf5 response to muscle damage. Morphologically an early acceleration of myotube formation was detected. In the *in vitro* model we also observed a reduction of myostatin expression in response to ectopic Dlk1, but combined with a significant reduction in MRF levels with exception of Myf5. The DLK1-C2C12 cells could not form myotubes, by adding antibodies this could be reversed even if the fiber forming cells still expressed Dlk1. The blocking ability of antibodies despite the intracellular presence of Dlk1 shows that the effect of Dlk1 most likely is based on extracellular presence and thus a paracrine or autocrine effect. Comparing the 2 models, the effect on myostatin is in common while that of the MRFs seems to differ. This indicates that the mechanisms by which Dlk1 influence the myostatin pathway can be segregated from that influencing the MRFs. The different effects of A and C2 isoforms could be due to the possibility that ovine Dlk1 do not exert all of Dlk1’s function in mice. However, a recent study showed that the C2 variant is associated with accelerated regeneration, a finding similar to ours [32] Even so, in the same study they also report that Dlk1-C2 stimulates differentiation and inhibits growth myoblasts, which is opposite to our results of normal growth combined with inhibition of differentiation under Dlk1-A enriched conditions. Therefore differential effects of Dlk1 isoforms should be taken into account. It should also be noted that we have used the CMV promoter and Waddell et al. [32] used the Myf-5 promoter in construction of the transgenic cell lines. Considering that Myf5 appears to be up regulated by Dlk1 this could influence the results.

In bone and adipose tissue Dlk1 has been shown to be an inhibitor of terminal differentiation [12] and our results with the A isoform points towards a similar function of this specific isoform in muscle. However other functions in muscle have been suggested recently such as support of self-renewal, acceleration of differentiation and muscle hypertrophy maintenance [32]. These apparently contradictory findings could be attributed to differential effects of the various Dlk1 splice variants, which opens the possibility that not only changes in amount of Dlk1 but also imbalance between isoform presentations could lead to a pathological condition.

### Dlk1 and the Myostatin Pathway

The growth inhibitor myostatin [22] was reduced on the mRNA level throughout regeneration in the TG mice, but the expression still appeared to be regulated in a pattern similar to the LC controls. Protein expression analysis corroborated this. This observation was intriguing in light of the mild muscle hypertrophy in the TG mice [8]. In DLK1-C2C12 cells myostatin expression was reduced by full-length Dlk1. Furthermore, DLK1-C2C12 cells showed continued proliferation as evidenced by the absence of myotubes, and reduced levels of *myoD* and *Myogenin* indicated suppressed differentiation. Thus ectopic expression of Dlk1 in C2C12 cells induced a phenotype reminiscent to myostatin null myoblasts [33]–[35].

In support of the direct relationship between myostatin and Dlk1, we observed an inverse regulation in the human myoblast cultures as well both on mRNA and protein level. Moreover, Dlk1 was predominantly expressed in quiescent G_0_ cells supporting a role for Dlk1 in self-renewal [32].

In our analysis of the myostatin pathway we observed that *Decorin* mRNA level was reduced while *Follistatin* mRNA level was increased in Dlk1 TG mice. Targeting of the myostatin signaling pathway could be an element in Dlk1 induced muscular hypertrophy due to an antagonizing effect of *Follistatin* [36] combined with a direct down regulation of *Myostatin* levels [26].

In the DLK1-C2C12 cells we observed no effect of full-length Dlk1 on *Follistatin* while *Decorin* and *Smad7* mRNA levels were up regulated. This suggests different effects of the Dlk1 isoforms on the myostatin pathway and that the effect of Dlk1 could be dependent on location. However, in the Dlk1 TG mice, ectopic Dlk1 was only expressed in myofibers and not in satellite cells thus any direct effect of Dlk1-C2 on the activated satellite cells and myoblasts was not detected.

Therefore, our results indicate that several possible regulatory pathways to control myostatin expression may exist.

### Rhabdomyosarcomas and Dlk1

MyoD activity and the balance between MyoD and Myf5 is essential for the myogenic program controlling terminal differentiation [37]. Targeting mechanisms that reduce the activity of MyoD thus result in a switch of rhabdomyosarcoma cells to a differentiated state [38]. Another factor employed in enhancing differentiation is myostatin. *In vitro* experiments investigating the effect of regulating myostatin in RMS cell cultures have shown that myostatin inhibits RMS cell proliferation [39] but also that reduction in myostatin enhance myogenic differentiation probably by improving MyoD function [40]. In this context our observation that Dlk1 is expressed in RMS is noteworthy, since Dlk1 both appears to reduce myostatin and change the Myf5-MyoD balance. Dlk1 in RMS was found in both undifferentiated cells and multinucleated, early differentiating structures as shown by co-localization with both Pax7 and Myogenin. In the RMS cell line experiments we found that Dlk1 was present during proliferation (A-204), could be induced (RD) or increased (A-204) by differentiation medium. Moreover, we detected the presence of both full-length soluble and membrane bound Dlk1 variants in the RMS cell lines. From this, Dlk1 could theoretically be part of an inhibitor of terminal differentiation in RMS, but also other suggested Dlk1 muscle functions could be active such as self-renewal. Addition of anti-Dlk1 antibody, however, did not change the differentiation potential of the RMS cell lines RD and A-204. A factor in this could be that sarcoma gene changes are much more complex, than regenerating cells, thus binding of Dlk1 may not be sufficient to induce differentiation. As the action of Dlk1 appears to be complex, imbalance between isoforms in addition to the specific quantity of Dlk1 might also play a critical role in RMS differentiation. However, the consistent Dlk1 expression in RMS shows that the dysregulation of the differentiation program blocks maturation in all types of RMS similar to the transient Dlk1 up regulation observed in other immature states e.g. fetal myogenesis [11].
